# 3D Printing of Mini Tablets for Pediatric Use

**DOI:** 10.3390/ph14020143

**Published:** 2021-02-11

**Authors:** Julius Krause, Laura Müller, Dorota Sarwinska, Anne Seidlitz, Malgorzata Sznitowska, Werner Weitschies

**Affiliations:** 1 Department of Biopharmaceutics and Pharmaceutical Technology, Center of Drug Absorption and Transport, Institute of Pharmacy, University of Greifswald, Felix-Hausdorff-Str. 3, 17487 Greifswald, Germany; julius.krause@uni-greifswald.de (J.K.); laura.mueller@uni-greifswald.de (L.M.); anne.seidlitz@uni-greifswald.de (A.S.); 2 Department of Pharmaceutical Technology, Medical University of Gdansk, 80416 Gdansk, Poland; d.sarwinska@gumed.edu.pl (D.S.); msznito@gumed.edu.pl (M.S.)

**Keywords:** 3D printing, FDM, pediatrics, mini tablets, caffeine, propranolol HCl

## Abstract

In the treatment of pediatric diseases, suitable dosages and dosage forms are often not available for an adequate therapy. The use of innovative additive manufacturing techniques offers the possibility of producing pediatric dosage forms. In this study, the production of mini tablets using fused deposition modeling (FDM)-based 3D printing was investigated. Two pediatric drugs, caffeine and propranolol hydrochloride, were successfully processed into filaments using hyprolose and hypromellose as polymers. Subsequently, mini tablets with diameters between 1.5 and 4.0 mm were printed and characterized using optical and thermal analysis methods. By varying the number of mini tablets applied and by varying the diameter, we were able to achieve different release behaviors. This work highlights the potential value of FDM 3D printing for the on-demand production of patient individualized, small-scale batches of pediatric dosage forms.

## 1. Introduction

Research in the field of 3D printing in recent years has shown how diverse the areas of application of this manufacturing technology are, particularly in the pharmaceutical field. Since the approval of the first oral 3D-printed dosage form Spritam in 2015, many other studies have been conducted regarding the applicability of 3D printing for the production of a wide variety of dosage forms [1,2,3]. Among all the additive manufacturing techniques that belong to the field of 3D printing, fused deposition modeling (FDM) is the most widely used method for oral dosage forms [1,2,3,4,5,6]. In this process, thermoplastic filaments are melted in a heatable and movable nozzle and deposited layer by layer onto a printing bed. This method offers the possibility to print many different shapes [5,7,8]. In addition, the filaments used can be loaded with active ingredient. Due to the flexibility and adaptability of the parameters of 3D printing, it is thus possible to produce patient-specifically dosed drugs [1,9,10,11,12,13].

This approach is particularly interesting for patient groups for whom there are not many drugs on the market. These include children in particular. Far fewer drugs are approved for these patients than for adults. The main development difficulties here are dose adjustment, taste masking, stability, and swallowability [14].

The lack of an appropriate dose often results in off-label use of drugs not approved for children. For this purpose, tablets can be divided by the physician or relatives, among others, whereby again the special requirements for off-label use and the divisibility of the drug must be observed. The problem with crushing the dosage form despite the fact that it is not intended to be divisible is that this often leads to overdosing or underdosing of the drug. In a position paper by the WHO from 2007 it is reported that the extent of the use of dosage forms in off-label use is not known, but that the use of off-label forms should be considered problematic and risky, which is why the development of suitable pediatric dosage forms should be promoted [15].

The manufacturing of tablets for pediatric use using 3D printing could be a way to produce precisely dosed dosage forms for children. Thus far, dose individualization in pediatrics is predominately achieved by producing hard capsules in community pharmacies. Studies have shown that the manufacturing process is susceptible to several influencing factors, which results in strong content fluctuations. For example, a study by Neumann and colleagues, who carried out content tests on 61 pediatric capsule formulations produced in 37 different pharmacies, showed that every fourth batch examined did not meet the requirements of the European Pharmacopoeia [16]. 

Many of the relevant pediatric drugs do not taste well. A sour, metallic, or bitter taste leads to a refusal of the child to take the medication [17]. For solid oral dosage forms in general, this problem can be solved by film coating. In contrast, when the drug is intended to be released in the oral cavity, flavoring agents must often be used to mask the bad taste. Taste masking can also be achieved through the use of melt extrusion. Hot melt extrusion (HME) is used in FDM printing to produce filaments. Advantages of this method for taste masking over the previously mentioned ones include the applicability of the method for water-soluble drugs, a continuous process design that is easy to scale up, and potential offering of good drug stability in the matrix [14,18,19,20,21].

Apart from the problematic situation with regard to dose-specific oral medications, there is also the difficulty in pediatric patients that children are often unable to swallow larger tablets or capsules. Possible solutions include 3D-printed orodispersible films, which are loaded with active ingredient and release it in the oral cavity so that swallowing is not necessary [4,22]. Furthermore, attempts have been made to improve the swallowability of the dosage form by producing chewable tablets that resemble candy in shape [23,24].

Another approach would be the printing of mini tablets, which have a diameter of <5 mm [25] and would therefore be easier to swallow. Mini tablets, in contrast to conventional tablets, are characterized by improved swallowability due to their small diameter and the dose can be changed more easily with the aid of the number of tablets administered [26,27]. Furthermore, the use of mini tablets in the field of pediatrics is well known [27,28]. As an example, Breitzkreutz and colleagues studied the swallowability and acceptance of mini tablets compared to syrup in infants and pre-school children and showed that the acceptability of mini tablets was superior to that of syrup [29,30].

To our knowledge, this approach of manufacturing mini tablets using FDM-based 3D printing has not yet been pursued. Therefore, the goal of this work was to investigate the suitability of FDM printing for the production of mini tablets. Different tablet diameters, various pediatrically relevant drug substances, and different polymers were investigated and characterized.

## 2. Results

Hot melt extrusion of drug-loaded filaments was successful at temperatures of 170 °C for the filaments based on hypromellose (HPMC) and at 140 °C for hyprolose (HPC) filaments. The filaments showed sufficient flexibility for further processing and a homogeneous diameter of about 2.8 mm. 

3D printing was used to produce mini tablets with a diameter of 1.5, 2.0, 3.0, and 4.0 mm. Each produced batch contained at least 200 mini tablets. To reduce negative printing effects such as stringing, we printed at least 10 mini tablets simultaneously per print run.

### 2.1. Uniformity of Dosage Units According to Ph. Eur. 2.9.40

The results of the test for mass variation according to the monograph 2.9.40 (Ph. Eur.) are shown in Table 1. Between 84.87% and 90.55% of the calculated content of caffeine was detected. Nevertheless, the results show that the tested samples of mini tablets of all diameters fulfilled the requirement of the European Pharmacopoeia in that the acceptance value must be below 15. Furthermore, the measured dimensions of the mini tablets containing HPMC and caffeine are also shown in Table 1.

### 2.2. Optical Appearance

**Figure 1:**
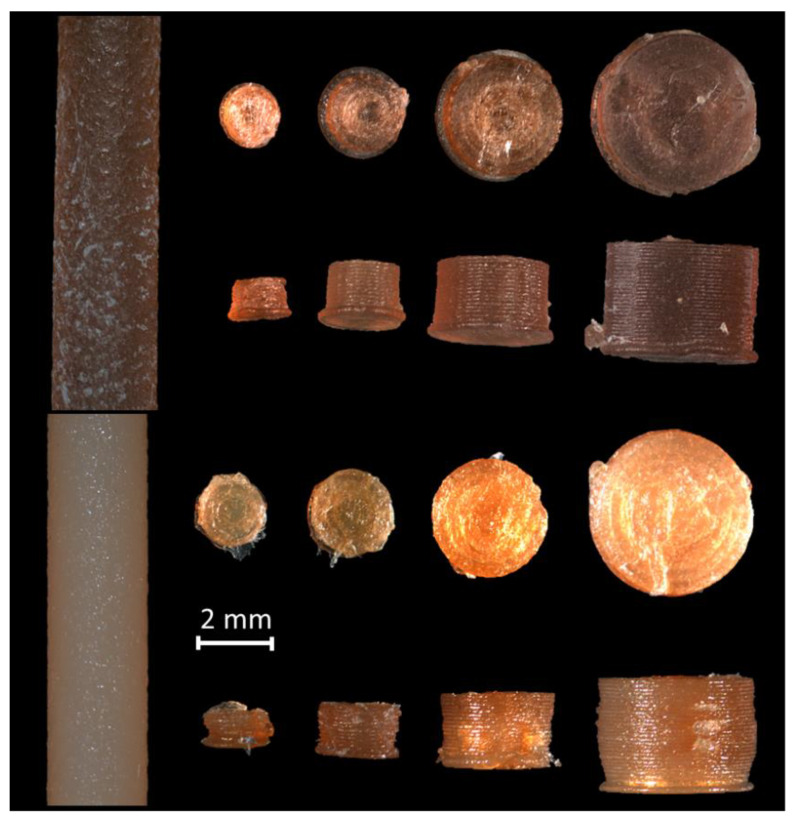
Microscopic images of the filaments and printed tablets with HPMC as polymer (top row: containing caffeine; bottom row: containing propranolol hydrochloride; from left to right: filament, mini tablet diameter 1.5, 2.0, 3.0, and 4.0 mm).

In Figure 1, the mini tablets with HPMC as polymer are shown. Both the caffeine and the propranolol hydrochloride containing HPMC tablets had a brownish color. While the mini tablets with a diameter larger than 2.0 mm had an even surface, the mini tablets with a diameter of 1.5 mm showed a slightly irregular shape.

The mini tablets containing caffeine showed a more homogenous surface and form compared to the mini tablets containing propranolol hydrochloride.

**Figure 2:**
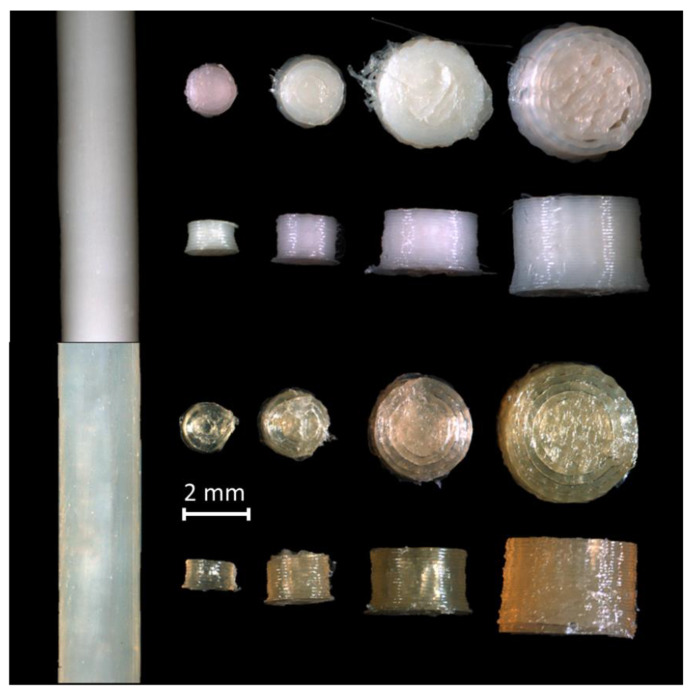
Microscopic images of the filaments and printed tablets with hyprolose (HPC) as polymer (top row: containing caffeine; bottom row: containing propranolol hydrochloride; from left to right: filament, mini tablet diameter 1.5, 2.0, 3.0, and 4.0 mm).

Figure 2 shows the 3D-printed mini tablets with HPC as polymer. As can be seen in the top row of Figure 2, the caffeine-containing dosage forms had a white, opaque coloration with a homogeneous surface. Small threads were occasionally visible on the dosage form.

The light-reflecting microscope images of the propranolol hydrochloride mini tablets are shown in the bottom row. Like the caffeine-containing mini tablets, the propranolol hydrochloride-containing mini tablets had an evenly shaped surface. As for the color, the mini tablets containing propranolol hydrochloride had a transparent, slightly yellowish color. The mini tablets with a diameter of 1.5 mm had a slightly irregular surface.

### 2.3. Scanning Electron Microscopy (SEM)

**Figure 3:**
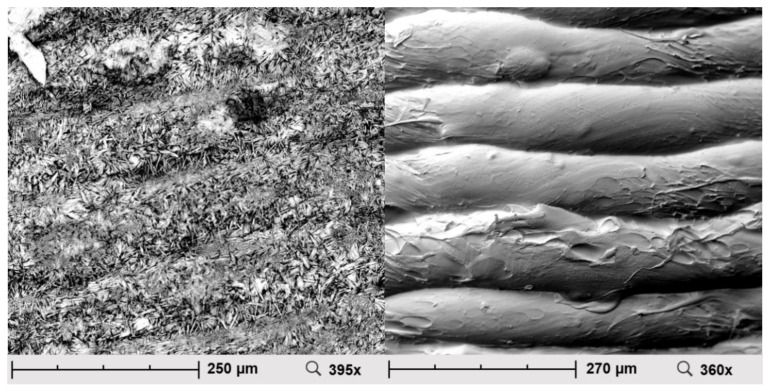
Scanning electron microscopic image of the mini tablets (left: caffeine-containing HPMC mini tablets, diameter 4.0 mm; right: propranolol hydrochloride-containing HPMC mini tablets, diameter 4.0 mm).

The electron micrographs of the mini tablets with HPMC as polymer are shown in Figure 3. For reasons of clarity, only one exemplary image per model drug is shown (the described observations were made in all printed mini tablets).

Needle-shaped entities were visible on the surface of the caffeine-containing mini tablets. In contrast, the surface of the propranolol hydrochloride-containing mini tablets appeared rather smooth and even, with isolated fiber-like structures.

**Figure 4:**
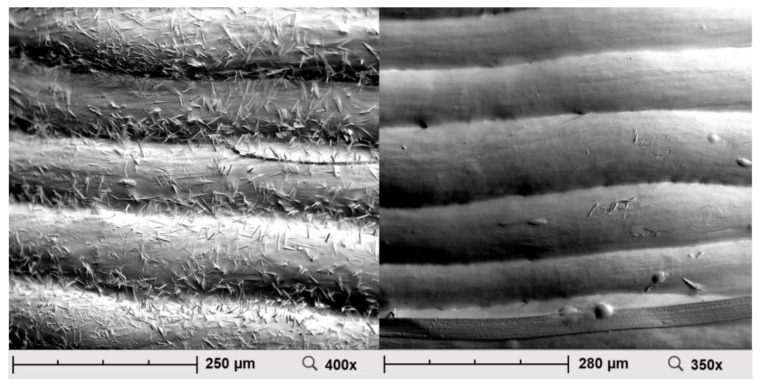
Scanning electron microscopic image of the mini tablets (left: caffeine-containing HPC mini tablets, diameter 4.0 mm; right: propranolol hydrochloride-containing HPC mini tablets, diameter 4.0 mm).

Figure 4 shows the SEM images of mini tablets with HPC as polymer. As observed previously, for the HPMC-based mini tablets, some needle-shaped entities were visible on the surface of caffeine containing dosage forms. The surfaces of HPC propranolol hydrochloride tablets appeared to be smoother than the surfaces of HPMC propranolol hydrochloride tablets and also showed no needle-shaped entities.

### 2.4. Thermal Analysis

**Figure 5:**
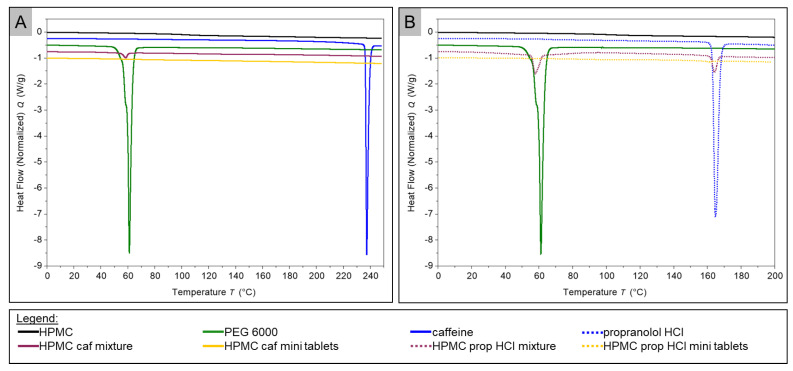
Differential scanning calorimetry (DSC) thermograms of HPMC-containing probes ((**A**) caffeine-containing probes; (**B**) propranolol hydrochloride-containing probes).

Differential scanning calorimetry (DSC) analysis was performed to determine the physical state of caffeine and propranolol hydrochloride in the printed tablets. The thermograms presented show the second heating cycle. Figure 5 depicts the thermograms of the caffeine, propranolol hydrochloride, PEG 6000, HPMC, the physical mixtures, and the milled mini tablets containing HPMC as polymer.

The thermogram of pure HPMC showed no endothermic peak but a step of the measurement curve was observed at about 98.5 °C. Furthermore, the thermogram of pure caffeine showed an endothermic event with a peak maximum at 237.26 °C (onset 236.54 °C). For pure PEG 6000, an endothermic peak at 61.16 °C (onset 59.51 °C) was monitored. The thermogram of pure propranolol hydrochloride showed an endothermic event at 164.85 °C (onset 162.96 °C). In the graph associated with the powder blend of the HPMC caffeine physical mixture, an endothermic peak can be seen at 58.57 °C (onset 55.01 °C). No peaks were detectable in the HPMC caffeine mini tablet thermogram. In the graph of the physical mixture of HPMC propranolol hydrochloride, two endothermic peaks were observed. The first one occurred at 57.93 °C (onset 55.61 °C), the second one at 164.24 °C (onset 162.09 °C). In the further course of the thermogram, the heat flow dropped drastically at temperatures above approximately 230 °C (for the sake of clarity, data not shown). The thermogram of the milled mini tablets containing propranolol hydrochloride revealed two endothermic events with a low amplitude. The first one was detected at 58.29 °C (onset 54.41 °C), the second one at 161.27 °C (onset 156.12 °C).

**Figure 6:**
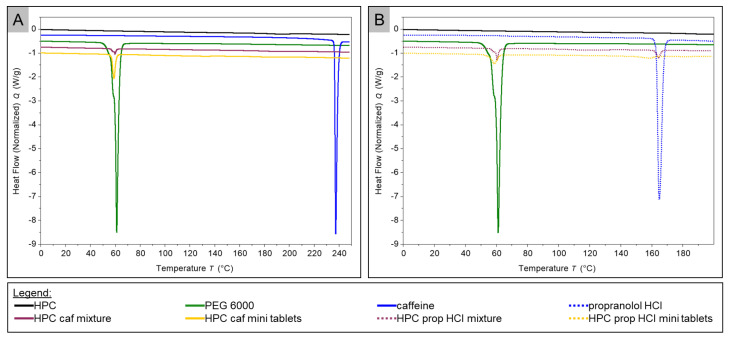
DSC thermograms of HPC-containing probes ((**A**) caffeine-containing probes; (**B**) propranolol hydrochloride-containing probes).

The thermograms of the HPC-based measurements are presented in Figure 6. The thermogram of pure HPC showed no endothermic peak. The analysis of the thermograms of the pure substances was already given previously. In the graph associated with the powder blend of the HPC caffeine batch, an endothermic peak occurred at 58.57 °C (onset 55.01 °C). The thermogram of the HPC caffein mini tablets showed on endothermic peak at 58.97 °C (onset 57.00 °C). In the graph of the physical mixture of HPC propranolol hydrochloride, two endothermic peaks were visible. The first one occurred at 57.93 °C (onset 55.61 °C), the second one at 164.24 °C (onset 162.09 °C). In the further course of the thermogram, the heat flow dropped drastically at temperatures above 230 °C (for the sake of clarity, data not shown). The thermogram of the milled mini tablets containing propranolol hydrochloride revealed two endothermic events. The first one was detected at 58.29 °C (onset 54.41 °C), the second one at 161.27 °C (onset 156.12 °C).

### 2.5. In Vitro Drug Release Studies

**Figure 7:**
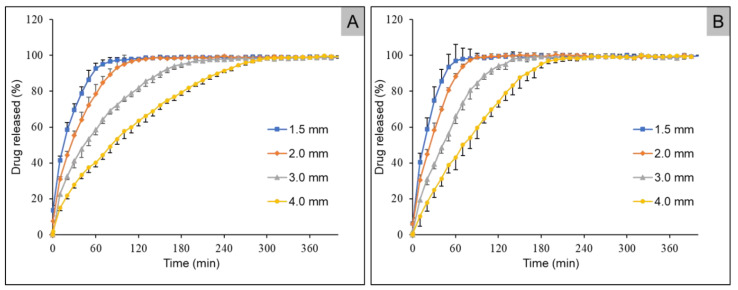
Dissolution profiles of HPMC mini tablets containing caffeine (**A**) and propranolol hydrochloride (**B**) with diameters of 1.5, 2.0, 3.0, and 4.0 mm (mean +/− SD, *n* = 5).

Figure 7 shows the dissolution profiles of the caffeine- and propranolol hydrochloride-containing mini tablets with HPMC as polymer. Mini tablets with smaller diameters showed faster dissolution. It was shown that the smaller the diameter, the faster the active ingredient was released. The mini tablets containing caffeine with a diameter of 4.0 mm released 80% of the active ingredient after about 190 min, while the mini tablets with propranolol hydrochloride released 80% of the active ingredient after only 140 min. This faster release behavior of caffeine and propranolol hydrochloride was also observed in the smaller mini tablets with a diameter of 3.0 mm. Here, 80% of the active ingredient was released after 120 min and after 80 min, respectively. Even for the tablets with smaller diameters, 80% of the caffeine was released after about 65 min, while 80% of the propranolol hydrochloride was released after 50 min. In the smallest 3D printed mini tablets with a diameter of 1.5 mm, 80% of the caffeine was released after 40 min, while 80% of the propranolol hydrochloride was released after about 35 min.

**Figure 8:**
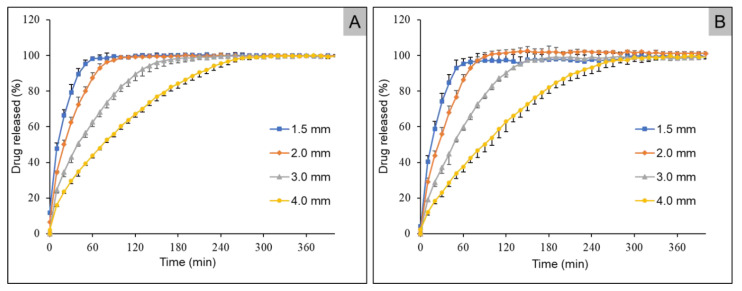
Dissolution profiles of HPC mini tablets containing caffeine (**A**) and propranolol hydrochloride (**B**) with diameters of 1.5, 2.0, 3.0, and 4.0 mm (mean +/− SD, *n* = 5).

In Figure 8, the release profiles of mini tablets containing HPC as polymer are given. A diameter-dependent release behavior was observed. As with the HPMC-containing mini tablets, the smallest tablets with a diameter of 1.5 mm showed the fastest release behavior. With both caffeine and propranolol hydrochloride, 80% of the active ingredient was released within 30 min. For the mini tablets with a diameter of 2.0 mm and 3.0 mm, 80% of the two drugs were released at the same time, namely, after 50 min and after 95 min. In the mini tablets with 4.0 mm, a slightly different release behavior of the two drugs was observed. While 80% of the caffeine was released after 160 min, 80% of the propranolol hydrochloride was released after 175 min.

## 3. Discussion

The aim of this work was to investigate the suitability of filament-based FDM 3D printing for the production of mini tablets with different diameters. For this purpose, two different model substances, caffeine and propranolol HCl, and two different polymers, HPMC and HPC, were investigated.

As raw material for the 3D printing, filaments with both active ingredients and both polymers were successfully produced by means of melt extrusion. Due to the different behavior of the polymers during melt extrusion, nozzles with different diameters were used to obtain a uniform filament diameter. This behavior has also been described in the literature [31,32]. The filaments with a theoretical drug content of 10% had a homogeneous diameter, uniform coloring, and sufficient flexibility. The drug loading can still be increased. The literature describes the production of filaments with an active ingredient content of up to 80% [33,34].

Subsequent 3D printing showed that FDM-based 3D printing is suitable for producing mini tablets with diameters between 1.5 and 4.0 mm. In order to optimize the optical appearance, we produced at least 10 tablets per print run. Depending on the diameter of the mini tablets, several hundred mini tablets can be produced in one print run.

### 3.1. Uniformity of Dosage Units According to Ph. Eur. 2.9.40

In addition to the general suitability of FDM-based 3D printing for printing mini tablets with diameters between 1.5 and 4.0 mm, the dimensional accuracy of the manufacturing process was also examined by examining the mass uniformity according to the monograph 2.9.40. It was found that the mini tablets of the investigated batch with caffeine as the active ingredient had a slightly reduced drug content compared to the theoretical API content. One reason for this could be the crystallization of the active ingredient, which was detected by electron microscopy, and the associated possibility of loss of active ingredient due to abrasion. Despite the slightly reduced active ingredient content, all mini tablets tested met the requirements of Ph Eur. It must be mentioned that, for reasons of feasibility and effort, the investigations described do not correspond to the specifications of the European Pharmacopoeia, since a test for content uniformity is recommend for the given active substance contents. Future studies should take this into account. The dimensions of the printed mini tablets largely corresponded to the specified parameters. Only the mini tablets with a diameter of 1.5 mm showed greater variations, which can also be seen in the optical characterization.

### 3.2. Optical Appearance

The examination of the optical appearance by using light microscopy images showed that the appearance was affected depending on the tablet diameter. It was found that the smaller the diameter of the mini tablets, the less accurate the printed shape. The smallest printed mini tablets with a diameter of 1.5 mm showed the most visually irregular surface. One reason for this is probably that the accuracy of FDM 3D printing is limited compared to other 3D printing methods due to the underlying technology of melting a polymer and subsequent extrusion through a nozzle. This limitation of FDM 3D printing is also sufficiently described in the literature [35,36]. More experiments with even smaller nozzle diameters and optimized pressure parameters as well as the use of other polymers can further improve the optical appearance of the mini tablets. The color change is due to thermal changes in the polymers, which also take place far below the decomposition temperatures. This is also described in the literature [37,38,39].

### 3.3. Scanning Electron Microscopy

The electron microscope images showed needle-shaped crystals on the surface of the caffeine containing mini-tablets when both polymers, HPMC and HPC, were used. With the composition of the mini tablets otherwise remaining the same, this observation could not be observed when using propranolol HCl as a model drug. This suggests that the crystals on the surface were caffeine. It was possible that caffeine was dissolved in the polymer during extrusion but recrystallized with increasing storage time. This process was also be observed macroscopically during storage of the caffeine-containing HPMC filament. This behavior has already been described for the drug caffeine in the literature [32].

### 3.4. DSC

DSC of caffeine (pure substance) showed an endothermic event, corresponding with the melting temperature described in the literature of about 238 °C for the pure drug [20]. Furthermore, the absence of a melting peak of caffeine in the DSC thermogram of 3D-printed mini tablets indicated that caffeine was dissolved in the HPMC. However, it should be noted that the melting peak was also missing in the physical mixture, indicating that the caffeine was dissolved in the polymer during the DSC investigation. Nevertheless, the scanning electron microscopic images showed that the active ingredient caffeine recrystallized from the printed tablets. This behavior cannot be confirmed by the DSC investigation. This is a disadvantage of thermal analysis methods such as DSC, in that during the heating process, which is analogous to thermal stress in hot melt extrusion and 3D printing, the active ingredient can potentially be dissolved in the polymer [40]. One analytical technique that has been used in the literature to determine the presence of the drug in the polymer is X-ray powder diffractometry (XRPD) [23,33,41]. In order to finally prove the thesis that caffeine recrystallizes, researchers should carry out crystallographic tests using X-ray powder diffractometry in further investigations.

For the HPC caffeine batches, a peak was found in the thermograms of the powder mixture and the milled tablets, which had a comparable position to the melting peak of the PEG 6000. Apart from this, no further peak was discernible in the further course of the two graphs. This observation allowed for the conclusion that the caffeine was dissolved in the polymer. In the powder mixture, a melting peak of the caffeine should be visible, but for reasons mentioned previously, it was missing in the thermogram.

The DSC measurement of pure propranolol HCl showed an endothermic event at about 165 °C, which was consistent with literature data for the melting point of the drug [42]. Above a temperature of about 200 °C, the active ingredient began to decompose, which can be recognized by a sudden sharp drop in the heat flow [43]. The thermograms of the HPMC tablets containing propranolol and the corresponding powder mixture each showed two peaks. In the powder mixture, these two peaks were strongly pronounced and were in similar positions to the melting peaks of the PEG 6000 and propranolol HCl. In the thermogram of the milled tablets, both peaks were much less pronounced. The endothermic event at about 60 °C had almost disappeared, and that at about 160 °C shifted a little to the left and was less pronounced. This can indicate that both substances had partially dissolved in the polymer. 

Comparing the thermograms of HPMC propranolol HCl tablets with those from HPC, we noticed that the observations were similar. Here, too, two peaks were found in both measurements, which were in the same position as those of the pure substances. As with the HPMC-based mini tablets, the peaks in the crushed tablets were smaller than in the measurement of the powder mixture. In addition, the melting peak of propranolol HCl was also shifted slightly to the left here. Here, too, the two substances did not appear to have dissolved completely in the matrix but were instead partially present in crystalline form.

### 3.5. Dissolution Studies

Depending on the diameter of the tablets, the mini tablets showed different release behavior. It was found that the mini tablets with a diameter of 4.0 m released their API the slowest. Since the two drugs and active ingredients used were BCS class 1 drugs and therefore were well soluble [44,45], it can be assumed that erosion and solubility of the carrier polymer were the main factors influencing the release from the dosage form. Despite the fact that the two drugs used were BCS class I drugs, there was a difference in the release behavior. When using HPMC as carrier polymer, propranolol hydrochloride was released faster. This trend was observed for all diameters investigated. A possible explanation for this could be a faster dissolution rate of propranolol hydrochloride in the dissolution medium.

This observation could not be made in mini tablets prepared from HPC. The release behavior of the two active ingredients was remarkably similar. Only in the tablets with the largest diameters was a faster release of caffeine present, with a 15 min time difference for 80% of drug release being observed. The different release behavior of the two drugs from the respective polymers could be based, among other reasons, on the fact that the release from HPC was slowed down with increasing swelling of the matrix due to impeded erosion compared to HPMC. Due to a higher viscosity of the swollen matrix of HPC, the drug release might occur mainly by diffusion. For the HPMC tablets, on the other hand, due to a lower viscosity of the swelling front, the release may have been controlled by a combination of diffusion and erosion.

In this work, 3D printing of mini tablets for use in pediatrics was investigated. The approach of using 3D printing to produce pediatric dosage forms has already been described in the literature. For example, Rycerz and colleagues used embedded 3D printing to produce gelatin-based Lego-like chewable bricks [24]. The variation of the appearance, the geometry, and the use of different materials were the main advantages. Another work by Scoutaris and colleagues also used 3D printing to produce dosage forms with geometries that were interesting for children [23]. The candy mix Starmix from the Haribo company was used as a model design. In contrast to the two aforementioned paper, pediatric dosage forms without a particularly child-friendly geometry were produced in the context of this work. Mini tablets offer the advantage in that, on the one hand, the use of mini tablets is established in pediatrics and, on the other hand, the dose and release behavior can be controlled by diameter and number. The process can also serve as an alternative to the previous, often error-prone production method of capsule manufacture.

## 4. Materials and Methods

### 4.1. Materials

Hypromellose (HPMC; Affinisol HME 15 LV) was kindly donated by Dow Chemicals (Midland, TX, USA). Hyprolose (HPC; Klucel ELF, Covington, KY, USA) was kindly gifted by Ashland (Covington, KY, USA). Caffeine and propranolol hydrochloride (Caelo, Germany) were used as model drug (Fagron, Germany). Caffeine was selected as one model drug substance because it is an API already described and established in melt extrusion and is also used in the treatment of apnoe of prematurity. Propranolol hydrochloride, which is used in the treatment of hemangiomas, was used as an example for a relevant drug. PEG 6000 (Merck, Germany) was used as plasticizer and fumed silica (Fagron, Germany) served as flow regulator.

### 4.2. Methods

#### 4.2.1. Preparation of Filaments Using Hot Melt Extrusion

Before the filaments were extruded, the powder mixtures were produced using a Turbula mixer (Turbula T2F, System Schatz, Germany, 49 rpm for 5 min). The composition of the batches can be found in Table 2 and Table 3.

After the mixing, the powder blends were processed into filaments using hot melt extrusion. A bench-top co-rotating, twin-screw extruder (Three-Tec ZE 12, Switzerland) with 12-mm diameter screws with an L/D of approximately 20:1, four heated zones, and a water-cooled feeding zone was used. The parameters of filament extrusion are shown in Table 4.

A 2.8 mm round-shaped die was used to extrude HPMC filaments. A larger nozzle with the diameter of 2.9 mm was used for the HPC filaments. The powder blends were fed into the extruder using a flat-tray feeder (Three-Tec ZD9FB, Switzerland; feed rate approximately 1.5%). A conveyor belt (Three-Tec AB17-21909, Switzerland) was used to collect and cool down the filament. The filament diameter was measured using a digital caliper (Powerfix, Kompernass Handels GmbH, Germany, accuracy ± 0.02 mm) to ensure homogenous filament diameter. To enable unproblematic 3D printing, we targeted a diameter of 2.8 mm. Filaments with variations in diameter of less than 0.05 mm were used. Filaments with greater variations were excluded from further testing.

#### 4.2.2. 3D Printing of Mini Tablets

The design of the mini tablets was based on a cylindrical shape. Four different sizes of mini tablets were printed for each batch. The dimensions of the mini tablets can be found in Table 5. The tablet geometry was designed with FreeCAD 0.18 and imported as a stereolithography file into the slicing software Cura (version 3.6.0, Ultimaker, The Netherlands). 

The mini tablets were produced using a standard fused deposition modelling printer Ultimaker UM 3 (Ultimaker, Netherlands). The printer is equipped with a Bowden-tube extruder and a hot end with a nozzle with a diameter of 0.25 mm. The printing settings can be found in Table 6. Blue painters’ tape was applied to the printing bed to improve first layer adhesion.

#### 4.2.3. Characterization of Mini Tablets

The mini tablets were characterized in terms of mass variation, optical appearance, thermal behavior, and dissolution behavior. 

#### 4.2.4. Uniformity of Dosage Units According to Ph. Eur. 2.9.40

For determination of mass uniformity according to Ph. Eur. 2.9.40. uniformity of dosage units, we randomly selected 10 mini tablets and weighed them individually using a digital analytical balance (Sartorius AX 124, Sartorius, Germany). Here, the test on mass variations was carried out as it is a non-destructive test. The average mass of the tablets was measured and the percentage deviation from the mean was determined. UV–VIS photometry was used for the determination of the drug content in a sample, which is needed for further calculation. The content tests were done in triplicate. For this purpose, the mini tablets were dissolved in phosphate buffer solution (pH 7.4) in 100 mL flasks. For the smallest tablets, due to small API concentration and thus very low absorption values, this study was performed in lower buffers volume (50 mL). The drug amount was measured via UV–VIS spectrometry with a fiber-optics-based system for on-line measurement (Cary 60, Agilent Technologies, Santa Clara, CA, USA, slit width: 10 mm, wavelength (caffeine): 272 nm, wavelength (propranolol hydrochloride): 290 nm). The test for mass uniformity was carried out on the mini tablets made from HPMC with caffeine as an exemplary batch. All 4 sizes were tested. Furthermore, the dimensions of the mini tablets were examined using taken using a reflected-light microscope (Zeiss Stemi 2000-C with Zeiss CL 1500 ECO, AxioCam and AxioVision software, all Carl Zeiss Microscopy GmbH, Oberkochen, Germany) with mini tablets containing HPMC and caffeine as an exemplary batch.

#### 4.2.5. Optical Appearance

Images of the extruded filaments and printed mini tablets were taken using a reflected-light microscope (Zeiss Stemi 2000-C with Zeiss CL 1500 ECO, AxioCam and AxioVision software, all Carl Zeiss Microscopy GmbH, Oberkochen, Germany). 

#### 4.2.6. Scanning Electron Microscopy (SEM)

The surface morphology of the printed mini tablets was examined using a scanning electron microscope (Phenom FP 3950/00, FEI Company, Hillsboro, OR, USA) with an acceleration voltage of 5 kV. Samples were placed on a metallic stub and gold–palladium coated under vacuum using a mini sputter Coater (SC7620, Quorum Technologies, Lewes, UK). Scanning electron microscope images were taken after 1 month of storage at room temperature.

#### 4.2.7. Thermal Analysis

To investigate the thermal behavior of the printed tablets, we examined samples with a conventional differential scanning calorimetry (DSC; DSC25, Texas Instruments, Dallas, TX, USA). Printed tablets were crushed using a batch mill (Tube Mill control 100, IKA, Staufen in Breisgau, Germany) before testing. Approximately 2 mg of the API, HPMC, HPC, PEG 6000, the physical powder mixtures, and crushed tablets were separately sealed in aluminum pans and heated with a rate of 10 °C/min in a temperature range from −40 to 250 °C. The temperature range was lowered to −40 to 200 °C for the measurement of propranolol-containing tablets due to thermal decomposition of the drug. Nitrogen was used as the purging gas with a flow rate of 50 mL/min. Each sample was subjected to a heat–cool–heat scan in order to measure and exclude the effect of moisture contents on filament plasticity and thermal ageing. The thermograms were analyzed with the Trios thermal analysis software (version 5.1.1, Texas instruments, Dallas, TX, USA).

#### 4.2.8. In Vitro Drug Release Studies

Drug release studies of printed tablets were carried out in 900 mL phosphate buffer 0.1 M USP (pH 7.4) using a basket apparatus (USP apparatus I, 37 °C, 75 rpm; Pharmatest DT 17, Pharma Test Apparatebau AG, Hainburg, Germany). In order to ensure comparability, we used a varying number of mini tablets to achieve an equal dose of the drug. A dose of approximately 10 mg caffeine/propranolol HCl was aimed for. Therefore, 2 mini tablets with a diameter of 4.0 mm, 5–7 mini tablets with a diameter of 3.0 mm, 15–20 mini tablets with a diameter of 2.0 mm, and 38–52 mini tablets with a diameter of 1.5 mm were used for the dissolution testing. Drug release was measured via UV–VIS spectrometry with a fiberoptics-based system for on-line measurement (Cary 60, Agilent Technologies, Santa Clara, CA, USA, slit width: 10 mm, measuring interval: 120 s, wavelength (caffeine): 272 nm, wavelength (propranolol hydrochloride): 290 nm). The release was scaled to the amount of drug measured in the plateau.

## 5. Conclusions

Within the scope of this work, drug loaded filament was successfully produced using hot melt extrusion. Furthermore, mini tablets containing caffeine and propranolol hydrochloride with diameters between 1.5 and 4.0 mm were successfully printed and characterized. Hereby, we could demonstrate that 3D printing of mini tablets enables dose individualization as well as variation of the release behavior depending on the size and the used polymer, and that the manufactured mini tablets fulfilled the requirements of the European Pharmacopoeia with regard to mass variation, despite the slightly uneven optical appearance of the tablets with the smallest diameters. This work highlights the potential value of FDM 3D printing for the on-demand production of patient individualized, small-scale batches of pediatric dosage forms.

## Figures and Tables

**Table 1:** Examination of mass variation for hypromellose (HPMC) caffeine mini tablets.

**Tablet Diameter (mm)**	4.0	3.0	2.0	1.5
**Measured diameter (mm)**	3.96	2.81	1.87	1.30
**Standard deviation of measured diameter (mm)**	0.07	0.01	0.03	0.10
**Measured tablet height (mm)**	3.01	1.91	1.49	0.93
**Standard deviation of Measured tablet height (mm)**	0.03	0.04	0.04	0.07
**Mean of mass (mg)**	41.70	17.49	5.63	2.47
**Standard deviation of mass (mg)**	1.98	0.47	0.31	0.34
**Mean of individual content, **$\bar{X}$ (%)	90.55	84.87	89.26	88.84
**Reference value, M (%)**	98.5	98.5	98.5	98.5
**Sample standard deviation, s**	0.05	0.19	0.24	0.19
**Sample size, *n***	10	10	10	10
**Acceptability constant, k**	2.4	2.4	2.4	2.4
**Acceptance value, AV**	8.06	14.10	9.38	10.13

**Table 2:** Composition of the powder mixtures containing HPMC (in % *w*/*w*).

Ingredients	Batch Name	
	HPMC-Caffeine	HPMC-Propranolol HCl
Caffeine	10	-
Propranolol HCl	-	10
Hypromellose	79.5	79.5
PEG 6000	10	10
Fumed Silica	0.5	0.5

**Table 3:** Composition of the powder mixtures containing HPC (in % *w*/*w*).

Ingredients	Batch Name	
	HPC-Caffeine	HPC-Propranolol HCl
Caffeine	10	-
Propranolol HCl	-	10
Hyprolose	79.5	79.5
PEG 6000	10	10
Fumed Silica	0.5	0.5

**Table 4:** Extrusion parameters.

Batch		HPMC-Caffeine HPMC-Propranolol HCl	HPC-Caffeine HPC-Propranolol HCl
Temperature	Zone 1 (°C)	120	90
	Zone 2–4 (°C)	170	140
Screw speed (rpm)		25	10

**Table 5:** Dimensions of the mini tablets.

Dimension	#1	#2	#3	#4
Diameter (mm)	4.0	3.0	2.0	1.5
Height (mm)	3.0	2.0	1.5	1.0

**Table 6:** Printing settings.

Setting	HPMC-Caffeine HPMC-Propranolol HCl	HPC-Caffeine HPC-Propranolol HCl
Printing temperature (°C)	200	170
Build plate temperature (°C)	60	60
Printing speed (mm/s)	30	30
Travel speed (mm/s)	30	30
Layer height (mm)	0.1	0.1
First layer height (mm)	0.17	0.17
Infill (%)	100	100
Print cooling fan	Enabled	Enabled
Filament retraction	Disabled	Disabled
Build plate adhesion	Skirt	Skirt

## Data Availability

The data presented in this study are available on request from the corresponding author.
